# Supplementary material for: Serological study of CoronaVac vaccine and booster doses in Chile: immunogenicity and persistence of anti-SARS-CoV-2 spike antibodies
Source: BMC Med. 2022 Jun 9;20:216. doi: 10.1186/s12916-022-02406-0 (PMC9177225; doi:10.1186/s12916-022-02406-0)
Supplement: Supplementary file 1 — Additional file 1: Fig. S1. Values of anti-SARS-CoV-2 spike IgG antibodies before immunization, and post-first and -second dose. [file 12916_2022_2406_MOESM1_ESM.pdf]

Lorem ipsum

Additional file 1: Fig. S1

Related to Figure 2. Values of anti-SARS-CoV-2 spike IgG antibodies before immunization, and post-first and -second dose.

	AUC <120	AUC 120-300	AUC >300
Pre-Vax Mean(SD)	54.42 ± 27.33	219.2 ± 53.04	960.9 ± 667.1
1st Dose +30d Mean(SD)	198.2 ± 192.5	458 ± 320.2	1523 ± 664.1
2st Dose +30d Mean(SD)	695.6 ± 372.2	682.6 ± 256.4	1517 ± 609.9
n	60	26	18
